# KIF11 inhibition for glioblastoma treatment: reason to hope or a struggle with the brain?

**DOI:** 10.1186/1471-2407-9-196

**Published:** 2009-06-22

**Authors:** Silvia Valensin, Chiara Ghiron, Claudia Lamanna, Andreas Kremer, Marco Rossi, Pietro Ferruzzi, Marco Nievo, Annette Bakker

**Affiliations:** 1 SienaBiotech S.P.A, Discovery Research, Strada di Petriccio Belriguardo 35, 53100 Siena, Italy

## Abstract

**Background:**

Glioblastomas (GBM) are typically comprised of morphologically diverse cells. Despite current advances in therapy, including surgical resection followed by radiation and chemotherapy, the prognosis for patients with GBM remains poor. Unfortunately, most patients die within 2 years of diagnosis of their disease. Molecular abnormalities vary among individual patients and also within each tumor. Indeed, one of the distinguishing features of GBM is its marked genetic heterogeneity. Due to the brain location of the tumor, the potential target inhibition for anticancer therapy must exhibit a manageable neurotoxicity profile in the concentration range in which the compounds show anti-proliferative activity.

Kinesin KIF11 inhibition by small molecules such as Monastrol or Ispinesib is currently under investigation in the field of malignant tumors. In the current study we have assessed the relevance of the anti-mitotic Kinesin-like protein KIF11 in human GBM cell-lines.

**Results:**

In this study the target was validated using a set of well characterised and potentially specific small molecule inhibitors of KIF11: an ispinesib analog, Monastrol, a Merck compound and 3 simplified derivatives of the Merck compound. Following an *in silico *selection, those compounds predicted to bear a favorable BBB permeation profile were assessed for their phenotypic effect on cell lines derived both from primary (U87MG) as well as treated (DBTRG-05-MG) glioblastomas. For some compounds, these data could be compared to their effect on normal human astrocytes, as well as their neurotoxicity on primary rat cortical neurons. The ispinesib analogue 1 showed an anti-proliferative effect on GBM cell lines by blocking them in the G2/M phase in a concentration range which was shown to be harmless to primary rat cortical neurons. Furthermore, ispinesib analog increased caspase 3/7-induced apoptosis in U87MG cells.

**Conclusion:**

In the area of cell cycle inhibition, KIF11 is critical for proper spindle assembly and represents an attractive anticancer target. Our results suggest that KIF11 inhibitors, when able to permeate the blood-brain-barrier, could represent an interesting class of anticancer drugs with low neurotoxic effects in the treatment of brain tumors.

## Background

Malignant gliomas, the most common subtype of primary brain tumors, are aggressive, highly invasive, and neurologically destructive tumors considered being amongst the deadliest forms of human cancers. The most widely used scheme for classification and grading of gliomas is that of the World Health Organization (WHO). Gliomas are graded on a scale from I to IV according to their degree of malignancy; the most aggressive being grade IV or Glioblastoma Multiforme (GBM).

The current study focused on GBM as it is considered the most common and most dramatic primary brain tumor in adults, with highest incidence in the elderly. Median survival for patients affected with GBM is only 9 to 15 months, and the majority of patients die within 2 years. The only -albeit moderately – successful currently used standard of care consists of a combination of surgery, chemo- and radiotherapy. Following surgery, patients are typically subjected to radiotherapy in combination with Temozolomide, an orally available DNA alkylating agent. Subsequently patients are further kept under Temozolomide treatment. Although there is no real difference in clinical benefit between patients with primary (de novo) or secondary (originally derived from low grade gliomas) GBMs [1], an impressive improvement of Temozolomide efficacy has been shown in patients expressing a methylated promotor of the methyl-guanidine-methyl transferase (MGMT) gene. The latter encodes for a DNA repair enzyme and is deemed responsible for a decreased Temozolomide DNA alkylating efficacy [2]. This limitation, together with the inherent, mechanism of action-linked toxicity of Temozolide also implies that the identification of better, molecular targeted therapies for the treatment of GBM remains.

In order to successfully eradicate GBM, a number of obstacles due to the location (the brain) and the nature (heterogeneous, infiltrating) of the tumor have to be overcome. GBMs do not only grow locally but infiltrate neighboring brain tissue through white matter tracts, perivascular, and periventricular spaces, and invading cells are often found centimeters away from the primary tumor mass [3]. The tumor's invasive nature is one of the cardinal features of malignant gliomas. This results in the inability of surgery to cure patients even when lesions arise in areas in which wide surgical resection would be possible. Chemotherapy should therefore be aimed at also affecting those tumor cells which are located in unresectable tumor areas. Since the blood-brain-barrier (BBB) could be expected to be intact in these areas, disease-modifying pharmacological intervention requires BBB-penetrating compounds.

Predicting central nervous system (CNS) partitioning remains a major challenge in drug design and needs to take a series of molecular properties into account already at the compound library design stage. *In vivo *experimental determination of blood-brain partitioning is difficult. It is time-consuming, expensive, and not suitable to screen large collections of chemicals or to assess the permeation of compounds at the beginning of the discovery process [4]. *In vitro *methods (passive artificial membrane permeability models, cellular monolayer models) are useful, although predictivity remains limited as the models cannot completely mimic the complexity of a dynamic *in vivo *system. Therefore computational (*in silico) *models have been developed in order to allow screening of large collections of compounds and to understand structure-activity relationships. From a target point of view, successful GBM treatment is hampered by the tumors cellular heterogeneity which features include proliferative, hypoxic and invasive cells.

Within the current study we decided to focus on the analysis of targets which affect GBM proliferation since in general anticancer drugs that perturb mitosis have been shown to play an important role in the therapy of malignant diseases. Targeted therapies under current investigation that are known to affect the cell cycle could be subdivided in three categories:

- Kinase inhibitors (mostly Cyclin-Dependent Kinases)

- DNA modifying agents (Alkylating agents, Topoisomerase inhibitors)

- Tubulin/microtubules modulators

Kinase inhibitors of the ATP-competitive sort are at risk of potential toxicity in relation to their potential lack of selectivity within the kinome. DNA modifying agents are known for their toxicity and/or drug resistance issues. Some tubulin/microtubule modulators show a significant effect on mitosis-independent cytoskeletal functions [5,6] such as maintenance of organelles, cell shape and intracellular transport phenomena. Since, especially neuronal processes highly depend on an intact cytoskeleton; the tubulin/microtubule modulator of choice should be such that only tumor mitosis is affected, leaving the mitosis-independent (and thus neuronal) functions untouched. Therefore, the potential value of kinesin inhibitors has been assessed. Kinesins are eukaryotic microtubule-associated motor proteins, which convert chemical energy released from nucleoside triphosphates (preferentially from ATP) into mechanical energy. There are over forty kinesins found in humans which all have a similar motor domain that uses ATP to perform its power stroke [7-9]. The difference between them is mostly determined by the nature of the adapters that attach the motor to the object that needs to be moved [10]. Kinesins can be classified according to function as either transport or mitotic kinesins. Transport kinesins play amongst others an essential role in the cytosolic movement and localization of synaptic vesicles in neurons and in physiological axonal transport processes [11].

Mitotic kinesins are involved in mitotic spindle assembly, maintenance and elongation, chromosome alignment and segregation, and microtubule depolymerisation, among other functions during cell division.

In this panorama, only the identification of highly specific and preferentially allosteric mitotic kinesin inhibitors which have the capacity to penetrate the BBB represent according to us a promising class of novel therapeutics for GBM.

## Results

### Descriptors calculation and BBB permeation prediction. Compound 1 is active and predicted to be BBB permeable

**Figure 1:**
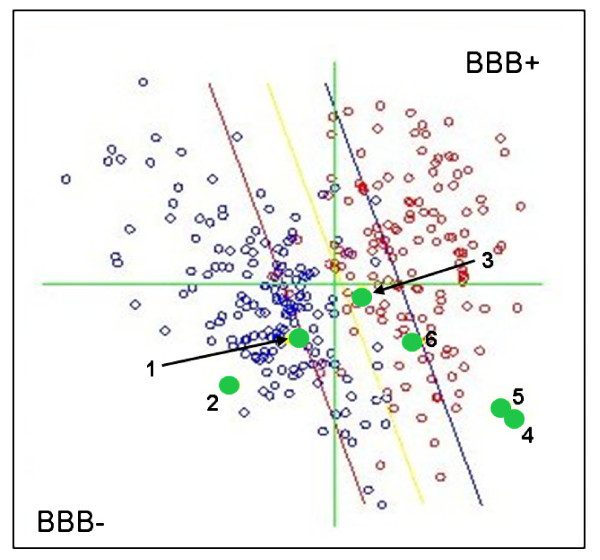
**Projection of the seven kinesin inhibitors onto the Volsurf global BBB model**. The blue circles at the left of the red line are those compounds belonging to the model and experimentally determined as BBB negative (non permeable) and the red circles above the blue line are those compounds which have been experimentally determined as BBB positive (permeable). Compounds projected within the area included between the red and the blue line (the uncertainty area) show a certain level of uncertainty and their BBB penetrating capacity should be cross validated using an additional model system.

Kinesin inhibitors (compounds **1–6**,) were analyzed in VolSurf for their CNS-partitioning properties, producing 94 molecular descriptors (grid spacing 0.5 Å) using three probes: a water probe (OH2) for hydrophilicity, a hydrophobic (DRY) probe computing the hydrophobic energy to be balanced with the hydrophilic energy computed by the OH2 probe, and a carbonyl oxygen probe (O) representing the hydrogen bond potential of the compounds. The DRY probe is a specific probe to compute hydrophobicity that can be estimated by means of the computation of three terms: the Lennard-Jones potential (that includes stacking, induction, and dispersion interactions with a maximum around -2.0 kcal/mol), the entropic (about -0.8 kcal/mol) and hydrogen-bonding contributions. The prediction of these seven compounds onto the global BBB model implemented in VolSurf resulted as shown in Figure 1.

In spite of classically unfavorable BBB characteristics (high molecular weight and high number of rotatable bonds) compound **1**, with a molecular weight of 583 and 10 rotatable bonds, was predicted by the Volsurf BBB global model to be in the "medium/undefinied" area of the graph. Since the Volsurf data were not decisive, a cross-validation using Cerius2 has been performed. This software predicted compound 1 to be BBB permeable. This second prediction is supported by the coumpound s detailed physico-chemical profile (Table 1), which shows a low number of hydrogen bond donor and acceptor functions, optimal lipophilicity and low polar surface area.

**Table 1:** Lipinski profile and BBB models prediction of the seven compounds

Compound number	MW	RBT	HBAcc	HBDon	PSA	AlogP	Cerius2 prediction	VolSurf prediction
1	583	10	5	1	56	5.6	**High penetrant (1)**	Medium/Undefined

2	355	5	3	3	53	2.9	Medium penetrant (2)	*Low permeant*

3	292	5	4	3	73	1.7	*Low penetrant (3)*	**High permeant**

4	255	2	2	1	26	4.4	**Very penetrant (0)**	**High permeant**

5	255	2	2	1	26	4.4	**Very penetrant (0)**	**High permeant**

6	294	4	3	0	41	2.9	**High penetrant (1)**	**High permeant**

Lipinski profile of the seven studied compounds and their predictions onto the BBB models of Cerius2 and Volsurf, based on their physico-chemical descriptors. Legend: MW = Molecular Weight; HBA = Hydrogen Bond Acceptors; HBD = Hydrogen Bond Donor; PSA = Polar Surface Area; AlogP = Negative logarithm of the octanol/water partition of the molecule.

In this sense, the results from VolSurf were confirmed and compound **1 **(in its neutral form) was predicted to have a higher probability of being BBB permeable (**bold**, Table 1) than compound **2 **(underlined, Table 1) in its neutral form. Compound **3 **was flagged (*italic*, Table 1) in the cross-validation method despite being a non-charged molecule at physiological pH, probably due to its unfavorable calculated physico-chemical properties (low AlogP, higher PSA).

All 'Merck fragments' (**4**, **5 **and **6**), were predicted to be CNS-penetrant according to both *in silico *models considered. Their potential for BBB penetrating capacity is also endorsed by their physico-chemical properties: they have a low molecular weight, a low number of rotatable bonds and few or no hydrogen bond donor/acceptor(s). The latter feature results in a reduction of the polar surface area and very acceptable values of lipophilicity when expressed as ALogP (Table 1).

Taken together, the physico-chemical features of **1 **and the prediction onto the BBB in silico models give higher chances for this compound to reach the brain; a calculated high lipophilicity together with a small polar surface area should increase the molecule's ability to cross the blood-brain barrier. It is of note that the combination of lipophilicity (here expressed as ALogP) and hydrogen-bonding (PSA) descriptors is a feature of many state-of-the-arts *in silico *BBB models [12].

### Ispinesib analogue 1 is anti-proliferative and significantly more effective than Monastrol (3) and Merk fragments (4 and 5)

The first stage of a study on the possibility of selectively inhibiting KIF11 in GBM with a brain entrant small molecule was to synthesize known inhibitors and to test against GBM cells.

**Figure 2:**
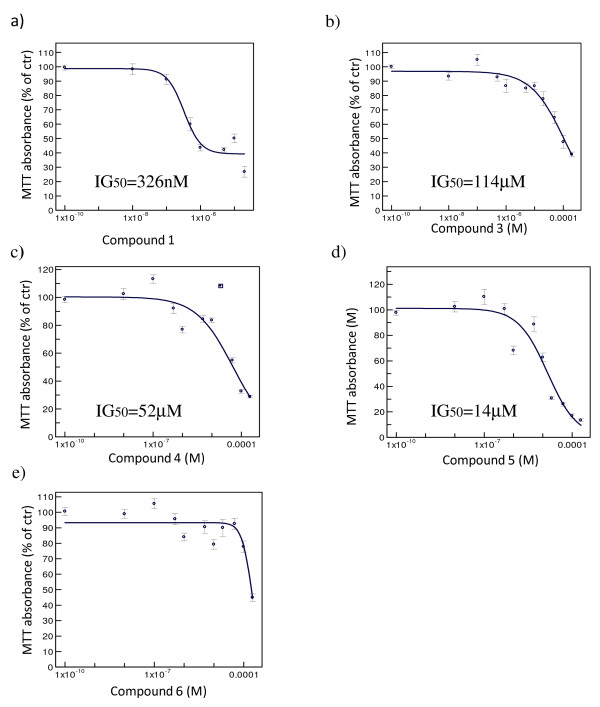
**Antiproliferative effect of KIF11 inhibitors on U87MG GBM cells**. All the panels a), b), c), d) and e) represented the concentration-response curve of the compounds **1**, **3**, **4**, **5 **and **6 **respectively. The cells were treated for 72 hours with increasing concentrations of the KIF11 inhibitor compounds **1**, **3**, **4**, **5 **and **6**. After the treatment the cell viability/proliferation were measured using the MTT assay. The analysis of the data was performed using XLfit4 software. The normalization is made taking the control as 100%. Each experiment was repeated three times at least in duplicate. Only compound **1 **showed an IG_50 _in a sub-micromolar range.

**Figure 3:**
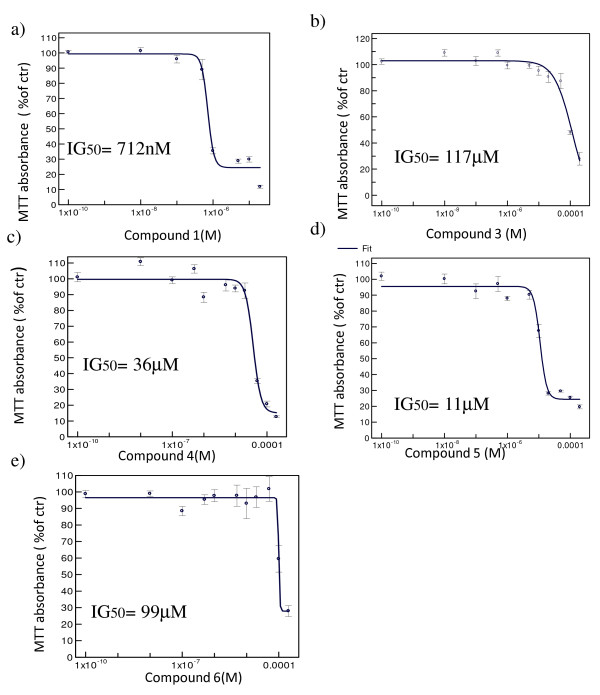
**Antiproliferative effect of KIF11 inhibitors on DBTRG-05-MG GBM cells**. All the panels a), b), c), d) and e) represented the concentration-response curve of the compounds **1**, **3**, **4**, **5 **and **6 **respectively. The cells were treated for 72 hours with increasing concentrations of the KIF11 inhibitor compounds **1**, **3**, **4**, **5 **and **6**. After the treatment the cell viability/proliferation were measured using the MTT assay. The analysis of the data was performed using XLfit4 software. The normalization is made taking the control as 100%. Each experiment was repeated three times at least in duplicate. Only compound **1 **showed an IG_50 _in a sub-micromolar range.

Compounds **1**, **3**, **4**, **5 **and **6**, which bear a positive *in silico *profile, were first tested for their antiproliferative effect on GBM cell-lines in an MTT proliferation assay: U87MG and DBTRG-05-MG cells were treated for 72 hours with the KIF11 kinesin inhibitors at concentrations varying from 10 nM to 200 μM, 24 h after seeding (compound **1 **from 10 nM to 20 μM, compound **3**, **4**, **5 **and **6 **from 10 nM to 200 μM). In these experiments, the media containing the compound was not changed during the whole incubation period. Doxorubicin, a well known anti-proliferative compound, was used as positive control (data not shown). The anti-proliferative activity was calculated as percentage of the remaining viable cells after treatment versus untreated cells. The results of these experiments are shown in figures 2 and 3. Ispinesib analog **1**, Monastrol **3 **and Merck fragments **4 **and **5 **induced a significant reduction in GBM cell proliferation, while compound 6 didn t seem active, even at the highest concentrations. However, Monastrol **3 **– analogously to what reported in the literature [13]- and both the Merck fragments (**4**-**5**) proved much less potent (Monastrol **3 **IG_50 _= 114 3M against U87MG and 117 3M against DBTRG-05-MG, Merck fragment **4 **IG_50 _= 52 3M against U87MG and 36 3M against DBTRG-05-MG and Merck fragment **5 **IG_50 _= 14 3M against U87MG and 11 3M against DBTRG-05-MG) than the Ispinesib analog (**1**) which showed an IG_50 _= 367 nM against U87MG and 712 nM against DBTRG-05-MG.

### Ispinesib analogue 1 induces G2/M cell cycle arrest and induces apoptosis

**Figure 4:**
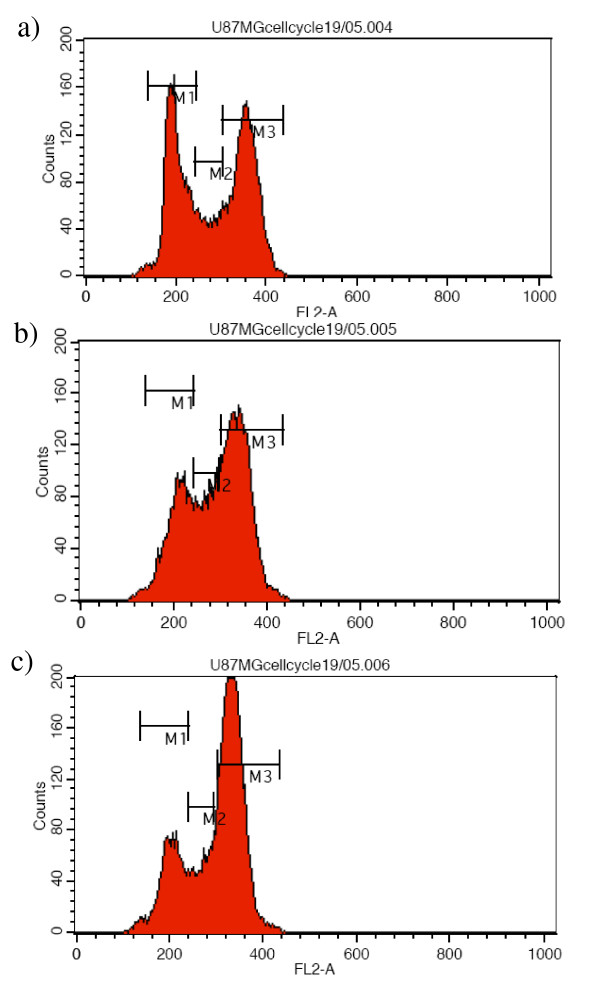
**Cell cycle profile on U87MG GBM cells**. Effect of the compound **1 **on the cell cycle of U87MG cells. The cells were un-treated (figure 4a), treated with 1 μM of the Ispinesib analog compound **1 **for 24 hours (Figure 4b) after 24 hours starvation. Nocodazole treatment was used as positive control (Figure 4c). Following incubation, the DNA of the cells was stained with propidium iodide and its content was analyzed by flow cytometry. Compound 1 induced a block of the cell cycle in G2/M phase. M1 was marker 1 and it represented G0/G1 phase; M2 was marker 2 and it represented the S phase; M3 was marker 3 and it represented G2/M phase.

**Figure 5:**
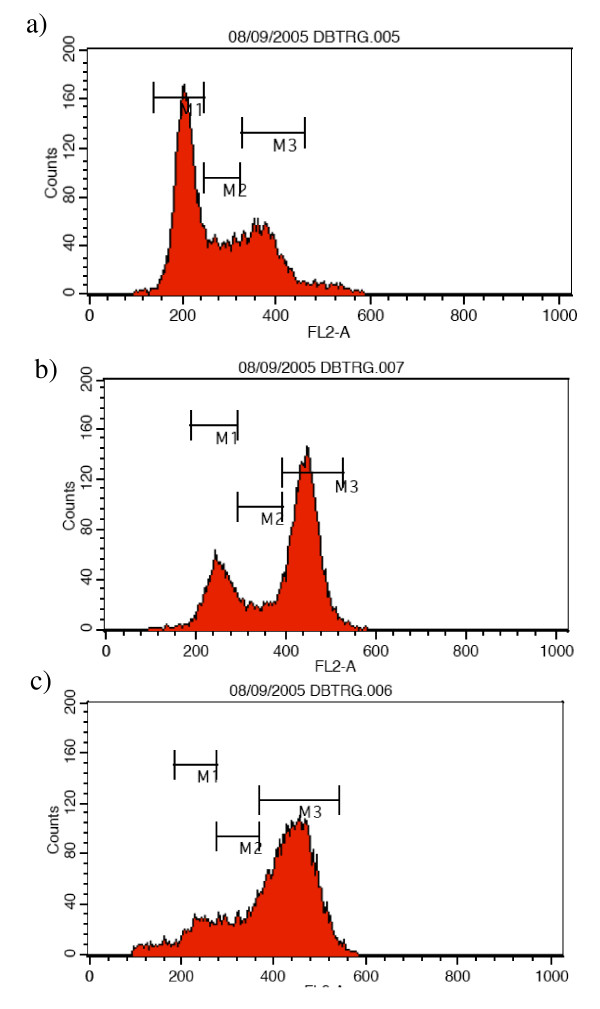
**Cell cycle profile on DBTRG-05-MG GBM cells**. Effect of the compound **1 **on the cell cycle of DBTRG-05-MG cells. The cells were un-treated (Figure 5a), treated with 1 μM of the Ispinesib analog compound 1 for 24 hours (Figure 5b) after 24 h starvation. Nocodazole treatment was used as positive control (Figure 5c). Following incubation, the DNA of the cells was stained with propidium iodide and its content was analyzed by flow cytometry. Compound 1 induced a block of the cell cycle in G2/M phase. M1 (marker 1) represented G0/G1 phase; M2 (marker 2) represented the S phase; M3 (marker 3) represented G2/M phase.

As it is known that KIF11 inhibitors induce a collapse of bipolar spindle with a consequent formation of a monopolar spindle resulting in a block of the cell-cycle [14], we assessed whether the Ispinesib analog **1 **affects the cell-cycle in GBM cell lines. Following 24 hours of serum deprivation in order to reach cell cycle synchronization, U87MG and DBTRG-05-MG cells were treated with Ispinesib analog compound **1 **at a fixed concentration of 1 μM for 24 hours. Nocodazole (a tubulin inhibitor known to block the cell-cycle in the G2/M phase) was used as a positive control. After incubation, the cells were fixed and stained with propidium iodide. Flow cytometry analysis of the cells showed that the Ispinesib analog **1 **had a potent effect on the cell-cycle. The treated cells (Figures 4b and 5b) demonstrated a significant increase in the amount of 4N DNA (G2/M = 61%) and subsequent decrease in 2N DNA (G1 = 22%) in comparison to the untreated cells (Figures 4a and 5a) (G2/M = 46% and G1 = 40%). These data are similar to those observed with Nocodazole (Figures 4c and 5c), and thus suggest that compound **1 **is able to induce a block in the G2/M phase in both cell-lines as a result of a failure in cytokinesis.

The data obtained indicated that compound **1 **induces a mitotic arrest in glioma cells, supporting its role as a potential anticancer target.

**Figure 6:**
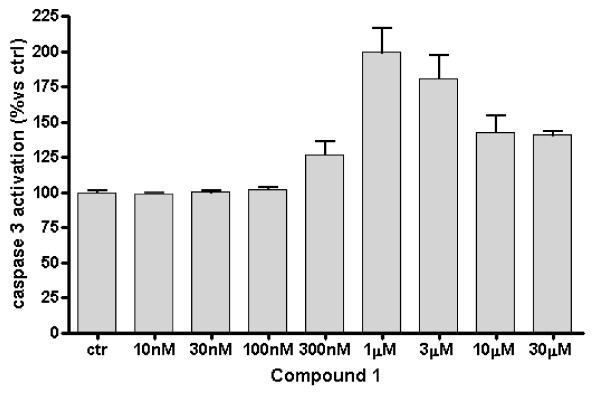
**Effect of the Ispinesib analog 1 on induction of apoptosis in U87MG**. The cells after cell adhesion were incubated for 24 hours with compound **1 **in a concentration range from 10 nM to 30 μM. The level of caspase 3 activation has been used as an indicator of apoptosis induction. The caspase 3 activation level was assessed after 24 hours of cell treatment with the compound at increasing concentrations (from 10 nM to 30 μM). The Ispinesib analog **1 **induced a 2-fold increase of caspase 3 levels, suggesting that this compound was a strong inducer of apoptosis in GBM cells.

In most cases, mitotic arrest induces apoptosis through mitochondrial membrane depolymerisation and caspase 3 activation. As kinesin inhibitors are known to increase caspase dependent apoptosis in a variety of tumor cell lines [5-16] the ability of compound **1 **to induce apoptosis in GBM cell lines was investigated. Caspase-3 activation level was thus assessed after 24 hours of treatment with compound **1 **at various concentrations (from 10 nM to 30 μM) against U87MG cells (Figure 6). Ispinesib analog **1 **induced caspase 3 activation starting from the concentration of 300 nM up to a maximal 2-fold increase of activity at 1 μM. The observed decrease at much higher concentration of **1 **(10 and 30 μM) could, in our opinion, be ascribed to a toxic effect. From these results we could conclude that this compound is a strong inducer of caspase 3-mediated apoptosis in U87MG cells.

### Ispinesib analogue 1 does not affect cell viability of human normal astrocytes and rat cortical neurons

**Figure 7:**
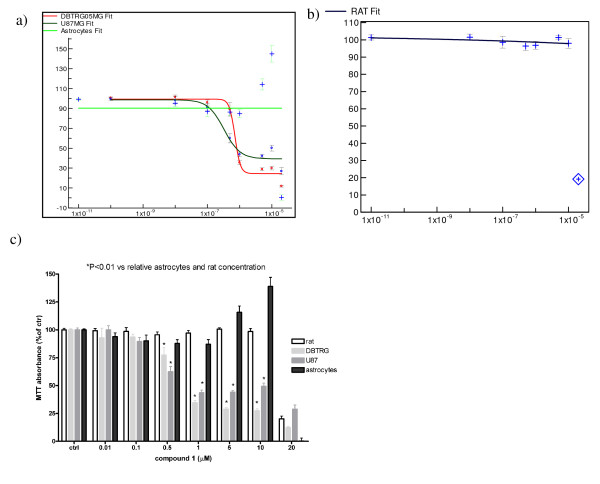
**Assessment of the therapeutic window of compound 1 on Human normal astrocytes and on rat cortical neurons vs U87MG and DBTRG-05-MG**. The cells were treated for three days with increasing concentrations of the compound **1**. Cell viability was assessed using MTT. From the overlay of the curves (panel a) we assessed the therapeutic window of compound **1 **by comparing cell viability of increasing concentrations of the compound (from 10 nM to 20 μM) on human normal astrocytes (green curve) versus GBM cell lines U87MG (black curve) and DBTRG-05-MG (red curve). The neurotoxicity was assessed testing compound **1 **on rat cortical neurons proliferation (panel b). In panel c the same experiment was reported as bar graph to underline the statistical differences at each concentration point. U87MG (gray bars) and DBTRG-05-MG (light gray bars) cells started to be statistically different from 500 nM and 1 μM respectively when compared to normal human astrocytes (black bars) and rat pure cortical neurons (white bars) where compound 1 was un-effective.

The potential for neurotoxic side effects of the Ispinesib analog **1 **was assessed by measuring its effect on normal human astrocytes viability. The cells were incubated for 72 hours with compound **1 **in concentrations ranging from 10 nM to 20 μM after seeding. The compound appears to have no effect at the concentration corresponding to U87MG and DBTRG-05-MG IG_50_. However, the compound becomes toxic to normal human astrocytes when its concentration exceeds 10 μM (Figure 7a). From these results we can conclude that compound **1 **could have an acceptable therapeutic window because in the concentration range [10 nM → 10 μM], the compound shows a good anti-proliferative effect on the glioma cell-lines without any interference with the normal human astrocytes behavior.

As proof that KIF11 inhibitors don't affect transport kinesin and that as such are specific compounds that block only the spindle formation and not the axonal transport, we tested compound **1 **in rat pure cortical neurons, which are non dividing cells. Typically, primary rat cortical neurons (after 12 days of maturation) were plated at a density of 300000 cells/well in 24 well plates. 24 hours after plating, cells were incubated for 72 hours with compound **1 **in concentrations ranging from 10 nM to 20 μM. The compound appeared to have the same behavior seen with normal astrocytes: the compound was toxic at concentrations exceeding 10 μM and no effect could be observed at the concentration corresponding to U87MG and DBTRG-05-MG IG_50 _(Figure 7b).

Both the human astrocytes and the rat cortical neuron results were confirmed by One Way Anova analysis (Tuckey's multiple comparison tests). In these experiments, starting from 500 nM, the overall viability of U87MG was significantly different from that of rat pure cortical neurons and normal human astrocytes (P value < 0.01), while DBTRG-05-MG viability started to be significantly different at 1 μM (P value < 0.01). At 20 μM there were no differences between glioma cell-lines, normal human astrocytes and rat pure cortical neurons (Figure 7c), probably due to the toxicity of the compound. From these results we could conclude that compound **1 **affects only GBM cell lines proliferation and not normal astrocytes proliferation, its therapeutic window appears suitable for future clinical applications. Furthermore, the compound affects only the KIF11 function and not the transport kinesins.

## Discussion

In the current study, mitotic kinesin KIF11, which is required for the separation of duplicated centrosomes and for the spindle formation [17], was deemed to be a promising target for glioblastoma treatment. The expression profile of KIF11 mRNA in glioblastoma cells versus normal astrocytes was first assessed. KIF11 mRNA expression is reported to be elevated in tumor samples compared with adjacent normal tissue in tumors derived from breast, colon, lung, ovary, rectum and uterus [18]. We confirmed this trend in GBM cell-lines (U87MG and DBTRG-05-MG) versus normal human astrocytes (data not shown).

Forward chemical genetics were applied to investigate the phenotypic effect of KIF11 inhibitors on GBM proliferation, apoptosis and cell cycle. This implied the selection BBB-permeating compounds known to specifically inhibit the target without affecting normal brain function.

To perform this study, we had access to a panel of existing preclinical efficacious KIF11 inhibitors. Monastrol (**3**), the first reported small molecule inhibitor, the quinazolinone derivative inhibitor from Cytokinetics SB-715992 and MKI-833, the Merck reported inhibitor (**2**) were demonstrated to be potent inhibitors of cell proliferation in several human tumor cell-lines (lung NCI-H460, A549; breast MDA-MB-231, MCF-7; colon HT29; ovarian SKOV-3, OVCAR-3; leukaemia HL-60, K-562, CNS SF-268; renal A498; osteosarcoma U2-OS; cervical HeLa) [14]. Ispinesib is being advanced to *Phase II clinical trials *as a general cancer therapeutic agent for cancers such as breast, ovarian and others. Moreover Monastrol (**3**) and other monastrol analogues are reported to be specific inhibitors of human GBM cells inducing growth inhibition and affecting spindle formation [13] without affecting the other kinesin-driven motor functions. Moreover, we selected a small set of Merck compound analogues whose smaller size meant they had greater probability of being brain penetrant. Since reaching the tumor in the brain was considered a critical criterion, only those compounds predicted to be BBB permeant were further investigated. The ability to pass the BBB is dependent on multiple factors, including lipophilicity, ionization profile, molecular size, polar surface area and molecular flexibility [19,20]. Relatively lipophilic drugs can cross the BBB by passive diffusion while polar molecules normally do not cross it unless they are substrates of specific active transport systems. There are several computational *in silico *tools that help chemists and biologists understand the complex physico-chemical features of compounds, and hence to predict the BBB properties of a molecule. In this study, two computational-statistical suites have been used for that purpose: VolSurf (VOLSURF, version 4.0; available from Molecular Discovery Ltd.: London, U.K. http://www.moldiscovery.com) and Cerius2 (Cerius2 version 4.11, available from Accelrys Inc. http://www.accelrys.com). These data provided a robust base for assigning the probability of compounds for crossing the BBB based on their physico-chemical profile. Only compounds **2**, **3**, **4**, **5 **and **6**, having passed the BBB selection filter, were further analyzed for their capacity to affect cell proliferation, to block the cell cycle and to induce apoptosis. We showed that the Ispinesib analogue compound **1 **(Figures 2 and 3) has a higher anti-proliferative activity against human GBM cell lines when compared to Monastrol (**3**) and to the Merk fragments (**4**, **5 **and **6**). The effect of compound **1 **on GBM cell-lines was also reflected by an increase of caspase 3 activity and by cell cycle block in G2/M phase.

In the neurotoxicity experiments carried against normal human astrocytes and rat cortical neurons, compound **1 **revealed to be characterized by a relatively broad therapeutic window. This could at least partially be attributed to compound **1 **selectivity for KIF11 over transport kinesins.

Monastrol (**3**), Ispinesib MKI-833 (**2**) and the majority of KIF11 inhibitors have been shown to have the same mechanism of action; they allosterically alter the ability of KIF11 to bind to microtubules and inhibit their movement by preventing the release of ADP without preventing the release of the KIF11-ADP complex from the microtubule [21]. This non-ATP binding, allosteric site, which is formed by helices α2 and α3 and Loop 5 appears to be specific for KIF11. However, several studies have shown that loop 5 mutations may induce resistance problems such as those demonstrated in colorectal cancer cells [22]. Should such mutability be identified in GBM patients as well, a need for KIF11 inhibitors that bind away from loop 5 may arise. The design of such novel KIF11 inhibitors, should take into account the recent evidence [23] that ATP-competitive compounds can/should not interfere with microtubule dynamics.

Overall, although specific KIF11 inhibitors are of great value to GBM, mechanism-based toxicity of kinesin inhibitors in general may limit the development of specific mitosis inhibitors.

## Conclusion

In this study we combined chemical, *in silico *ADME and PK properties and biological approaches to analyze the effect of a pannel of mitotic kinesin KIF11 inhibitors on Glioblastoma cell lines. Following an *in silico *selection for BBB penetration, KIF11 inhibitors were analysed for their effect on cell proliferation, cell-cycle and apoptosis induction. The Ispinesib analog **1**, which resulted able to not only affect cell proliferation, but also block cell cycle and induce apoptosis, was tested in normal human astrocytes and in rat pure cortical neurons to evaluate its therapeutic window and neurotoxicity. Although specific KIF11 inhibitors demonstrating a broad therapeutic window could be of great value for the treatment of GBM, the design of these compounds is hampered by high homology between motor and mitotic kinesins.

## Methods

### Physico-chemical descriptors

VolSurf is molecular modeling software that generates 2D molecular descriptors from 3D molecular interaction field (MIF) on GRID maps [24]. VolSurf compresses the information contained in 3D maps calculated by GRID into a predefined set of 2D numerical descriptors that can be interpreted in terms of structure. The GRID force field uses a potential based on the total energy of interaction [the sum of Lennard-Jones (E_LJ_), H-bonding, electrostatic (E_HB_) and hydrophobic terms (E_Q_) added to the Entropic term (S)] between a target molecule and a probe.

VolSurf was used to calculate the principal molecular physico-chemical properties, translated into descriptors within statistical analyses and it is specifically designed for the optimization of *in silico *ADME and pharmacokinetic properties for pharmaceutically relevant compounds [24].

The cross-validation in this study was performed with Cerius2, a software providing tools for drug design similar to VolSurf, but based on different algorithms for statistical analysis, calculation of descriptors and prediction onto global models. In this case, the Cerius2 global BBB model was used for cross-validating the predictions in VolSurf.

### Compounds synthesis

**Figure 8:**
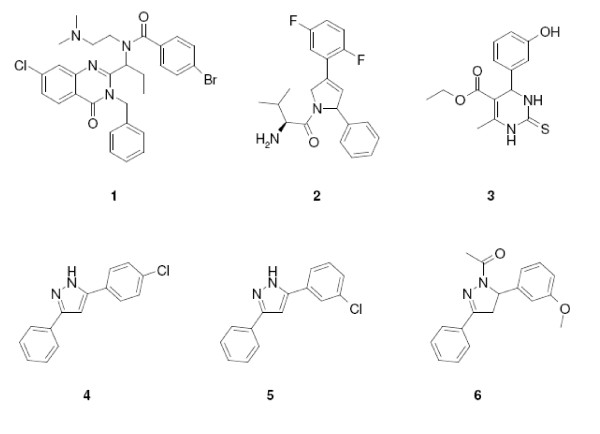
**Chemical structures of the compounds used**. the analogue of Ispinesib **1**, analogue of Merck compounds **2**, Monastrol **3 **and **4**, **5**, **6 **which could be considered as fragments of compound **2**.

The Ispinesib analogue compound **1 **(Figure 8) was synthesized following slight modifications from the reported literature (WO200198278 A1). Monastrol, compound **3**, is commercially available from Sigma-Aldrich. A small subset of compounds was also selected from our compound collection which could be considered as 'fragments 'of Merck compound, compound **2**, and further described as 'Merck fragments'. These compounds are **4**, **5**, and **6 **(as shown in Figure 8). Merck compound, compound 2, was obtained by Ferrara University with a few modifications from the literature ([25] and WO2004087050).

### Glioma cells

GBM cell lines examined, U87MG (derived from a de novo GBM) and DBTRG-05-MG (derived from recurrent GBM), were both obtained from Interlab Cell Line Collection (Genova, Italy). U87MG and DBTRG were cultured in RPMI containing 10% heat inactivated fetal bovine serum (Fbs), 2 mM Glutamax, 100 units/ml Penicillin and 100 μg/ml Streptomycin. For this study were also used primary cultures of rat pure cortical neurons obtained from embryonic day (E) 12 rat embryos according to a well established method that allow the growth of a >99% pure neuronal population [26]. Normal human astrocytes were obtained from Lonza. The primary cultures of rat pure cortical neurons were grown in neurobasal medium supplemented with B27 in 24-well plates. All cell types were maintained in water saturated atmosphere (5% CO_2_) at 37°C.

### Proliferation assay

Cell proliferation was measured using MTT [3-(4,5-dimethylthiazol-2-yl)-2,5-diphenyltetrazolium bromide, SIGMA] -based assay. U87MG and DBTRG-05-MG were plated at a density of 10000 and 20000 cells per well, respectively, normal human astrocyte and rat cortical neurons at a density of 30000 cells per well in 24-well tissue culture plate in a total volume of 500 μL medium per well. After cell adhesion the medium was replaced with medium containing different concentrations range of compound **1**, from 10 nM to 20 μM, or compounds **3**, **4**, **5 **and **6**, from 10 nM to 200 μM. Following three days of incubation, the medium was replaced by 250 μL fresh medium without Fbs and 25 μL of MTT solution 5 μg/μl were added to each well. After an incubation time of 4 hours at 37°C, the formazan crystals formed by the metabolically active cells were solubilized by addition of 250 μl solution constituted by isopropanol, Triton X-100 and HCl. The 24 well plates were shaken for 10 min on a microplate shaker and absorbance at 570 nm was measured using a plate reader [Victor III, Perkin Elmer].3 Background absorbance at 690 nm was subtracted before data analysis. The anti-proliferative activity was calculated as percentage of remaining viable cells after treatment versus control untreated cells. Each experiment was performed at least in duplicate in three different experiments. Data analysis was performed using Excell Fit software.

### Cell-cycle flow cytometry

Cell cycle distribution after compound treatment was determined by measuring the amount of cellular DNA using propidium iodide staining. U87MG and DBTRG-05-MG cells were plated at a density of 1000000 in a T75 flask; the day after cells were synchronized in serum free conditions for 24 h and then incubated for 24 hours with a fixed concentration (1 μM) of compound **1 **or Nocodazole. Following incubation, the cells were harvested and fixed with cold ethanol, washed with PBS, treated with 10 mg/ml RNAse for 15 minutes, and incubated with 50 μg/ml propidium iodide for 15 minutes. DNA content in different phases of cell cycle was determined using flow cytometry (FACScalibur, BD Biosciences Immunocytometry System) measuring propidium iodide emission at 580 nm. Cell cycle distribution was analyzed using BD CellQuest™ Pro software (BD Biosciences Immunocytometry System).

### Apoptosis Assay

The effect of the compound **1 **on apoptosis was tested using a time resolved fluorescence technology based on the TruPoint™ Caspase-3 Kit^96 ^(PerkinElmer, USA). This kit is based on the measurement of increased caspase 3/7 activity. When active, caspase 3 cleaves the substrate Z-DEVD and forms aminoluciferin, which is in turn a substrate for luciferase. The cells were plated at a density of 10000 cells per well in white ViewPlate™-96 wells (PerkinElmer, USA) and after cell adhesion they were incubated for 24 hours with compound **1 **in a concentration range from 10 nM to 30 μM. Subsequently the cells were washed and total cellular protein extract was prepared. Specific substrate and detection buffer were added and the luminescence was measured. (Victor III, PerkinElmer). Induction of apoptosis was evaluated as percentage of caspase 3 activation in treated versus control (untreated) cells. Each experiment was performed in triplicate in three different experiments.

## Abbreviations

(GBM): Glioblastoma; (WHO): World Health Organizzation; (BBB): Blood Brain Barrier; (CNS): Central nervous system; (ANOVA): Analysis of variance.

## Competing interests

The authors declare that they have no competing interests.

## Authors' contributions

SV performed all the proliferation experiments together with PF, cell-cycle profile and wrote the manuscript with the coordination and the assistance of AB and PF; CG conducted the compounds synthesis; CL performed the drug profile study; MR conducted the apoptosis experiments and AK performed the structural analysis of the compounds. MN provided an analysis of the cell-cycle targets actively being investigated for clinical purposes. All the authors read and approved the final manuscript.

## Pre-publication history

The pre-publication history for this paper can be accessed here:

http://www.biomedcentral.com/1471-2407/9/196/prepub

## References

[B1] Maher EA Furnari FB Bachoo RM Rowitch DH Louis DN Cavenee WK DePinho RA Malignant glioma: genetics and biology of a grave matter Genes & Development 2006 15 1311 1333 10.1101/gad.891601 11390353

[B2] Bent MJ Van den The role of chemotherapy in brain metastasis European Journal of Cancer 2003 39 2114 2120 10.1016/S0959-8049(03)00577-X 14522368

[B3] Hochberg FH Pruitt A Assumptions in the radiotherapy of glioblastoma Neurology 1980 9 907 11 10.1212/wnl.30.9.907 6252514

[B4] Lombardo F Blake JF Curatolo WJ Computation of brain-blood partitioning of organic solutes via free energy calculations J Med Chem 1996 24 4750 5 10.1021/jm960163r 8941388

[B5] Weaver BAA Cleveland DW Decoding the links between mitosis, cancer, and chemotherapy: the mitotic checkpoint, adaptation, and cell death Cancer Cell 2005 8 7 12 10.1016/j.ccr.2005.06.011 16023594

[B6] De Bonis S Skoufias DA Lebeau L Lopez Robin G Margolis RL Wade RH In vitro screening for inhibitors of the human mitotic kinesin Eg5 with antimitotic and antitumor activities Molecular Cancer Therapeutics 2004 3 1079 1090 15367702

[B7] Goodson HV Kang SJ Endow SA Molecular Phylogeny of the kinesin family of microtubule motor proteins Journal of Cell Science 1994 107 1875 1884 7983154 10.1242/jcs.107.7.1875

[B8] Vale RD Fletterick RJ The design plan of kinesin motors Annual Review of Cell and Developmental Biology 1997 13 745 777 10.1146/annurev.cellbio.13.1.745 9442886

[B9] Hirokawa N Kinesin and dynein superfamily proteins and the mechanism of organelle transport Science 1998 279 519 526 10.1126/science.279.5350.519 9438838

[B10] Kull FJ Motor proteins of the kinesin superfamily: structure and mechanism Essays Biochem 2000 35 61 73 12471890 10.1042/bse0350061

[B11] Barry M Millecamps S Julien J Garcia M New movement in neurofilament transport, turnover and disease Exp Cell Res 2007 313 2110 20 10.1016/j.yexcr.2007.03.011 17451679

[B12] Clark DE In silico prediction of blood brain barrier permeation Drug Discov Today 2003 8 927 933 10.1016/S1359-6446(03)02827-7 14554156

[B13] Muller C Gross D sarli V Gartner M Giannis A Bernhardt G Buschauer A Inhibitors of Kinesin EG5: antirpoliferative activity of monastrol analogues human glioblastoma cells Cancer Chemother Pharmacol 2007 59 157 164 10.1007/s00280-006-0254-1 16703323

[B14] Coleman PJ Fraley ME Inhibitors of the mitotic kinesin spindle protein Expert Opin Ther Patents 2004 12 1659 1667 10.1517/13543776.14.12.1659

[B15] Tao W South VJ Zhang Y Davide JP Farrell L Kohl NE Sepp-Lorenzino L Lobell RB Induction of apoptosis by an inhibitor of the mitotic kinesin KSP requires both activation of the spindle assembly checkpoint and mitotic slippage Cancer Cell 2005 8 49 59 10.1016/j.ccr.2005.06.003 16023598

[B16] Tao W South VJ Diehl RE Davide JP Sepp-Lorenzino L Fraley ME Arrington KL Lobell RB An inhibitor of the kinesin spindle protein activates the intrinsic apoptotic pathway independently of p53 and de novo protein synthesis Molecular and Cellular Biology 2007 27 689 698 10.1128/MCB.01505-06 PMC1800817 17101792

[B17] Brier S Lemaire D De Bonis S Forest E Kozielski Identification of the protein binding region of S-trityl-L-cysteine, a new potent inhibitor of the mitotic kinesin Eg5 Biochemestry 2004 43 13072 13082 10.1021/bi049264e 15476401

[B18] Koller E Propp S Zhang H Zhao C Xiao X Chang M Hirsch S Shepard P Koo S Murphy C Glazer R Dean N Use of chemically modified antisense oligonucleotide library and validate Eg5 (Kinesin-like 1) as a target for antineoplastic drug development Cancer Research 2006 66 2059 2066 10.1158/0008-5472.CAN-05-1531 16489005

[B19] Abraham MH Chada S Mitchell R Hydrogen Bonding 33 Factors that Influence the Distribution of solutes between blood and brain J Pharm Sci 1994 83 1257 1268 10.1002/jps.2600830915 7830242

[B20] Crivori P Cruciani G Carrupt PA Testa B Predicting Blood-Brain barrier Permeation from Three-Dimensional Molecular Structure J Med Chem 2000 43 2204 2216 10.1021/jm990968+ 10841799

[B21] Lad L Luo L Carson JD Wood KW Hartman JJ Copeland RA Sakowicz Mechanism of inhibition of human KSP by ispinesib Biochemistry 2008 47 11 3576 85 10.1021/bi702061g 18290633

[B22] Jackson JR Auger KR Gilmartin AG Eng WK Luo L Concha N Sutton D Diamond M Giardiniere M Zhang S Belmont L Lee Y Anderson R Wood KW Sakowicz R Huang PS GlaxoSmithKline, 1Collegeville PA C207 A Resistance Mechanism for the KSP Inhibitor *ispinesib *Implicates Point Mutations in the Compound Binding Site 2005 Cytokinetics, South San Francisco, CA [Molecular targets and cancer theraputics AACR-NCI-E]

[B23] Knight SD Parrish CA Recent progress in the identification and clinical evaluation of inhibitors of the mitotic kinesin KSP Curr Topics Med Chem 2008 8 888 904 10.2174/156802608784911626 18673173

[B24] Cruciani G Crivori PA Carrupt PA Testa B Molecular Fields in Quantitative Structure-Permeation Relationships: The VolSurf approach J Mol Struct 2000 503 17 30

[B25] Fraley ME Garbaccio RM Arrington KL Hoffman WF Tasber ES Coleman PJ Buser CA Eileen S Walsh ES Hamilton K Christine Fernandes C Michael D Schaber MD Lobell RB Tao W South VJ Yan Y Kuo LC Prueksaritanont T Shu C Torrent M Heimbrook DC Kohl NE Huber HE Hartman GD Kinesin spindle protein (KSP) inhibitors. Part 2: The design, synthesis, and characterization of 2,4-diaryl-2,5-dihydropyrrole inhibitors of the mitotic kinesin KSP Bioorganic & Medicinal Chemistry Letters 2006 16 1775 79 10.1016/j.bmcl.2006.01.030 16439123

[B26] Copani A Condorelli F Caruso A Vancheri C Sala A Giuffrida SAM Canonico PL Nicoletti F Sortino MA Mitotic signaling by beta-amyloid causes neuronal death FASEB J 1999 13 2225 2234 10593870

